# Supplementary material for: Laser Synthesized Core-Satellite Fe-Au Nanoparticles for Multimodal In Vivo Imaging and In Vitro Photothermal Therapy
Source: Pharmaceutics. 2022 May 5;14(5):994. doi: 10.3390/pharmaceutics14050994 (PMC9144942; doi:10.3390/pharmaceutics14050994)
Supplement: Supplementary file 1 [file pharmaceutics-14-00994-s001.zip › pharmaceutics-1685755-supplementary.pdf]

pharmaceutics

Supplementary Materials: Laser Synthesized Core-Satellite Fe-Au Nanoparticles for Multimodal In Vivo Imaging and In Vitro Photothermal Therapy

Olga Yu. Griaznova, Iaroslav B. Belyaev, Anna S. Sogomonyan, Ivan V. Zelepukin, Gleb V. Tikhonowski, Anton A. Popov, Aleksei S. Komlev, Petr I. Nikitin, Dmitry A. Gorin, Andrei V. Kabashin and Sergey M. Deyev

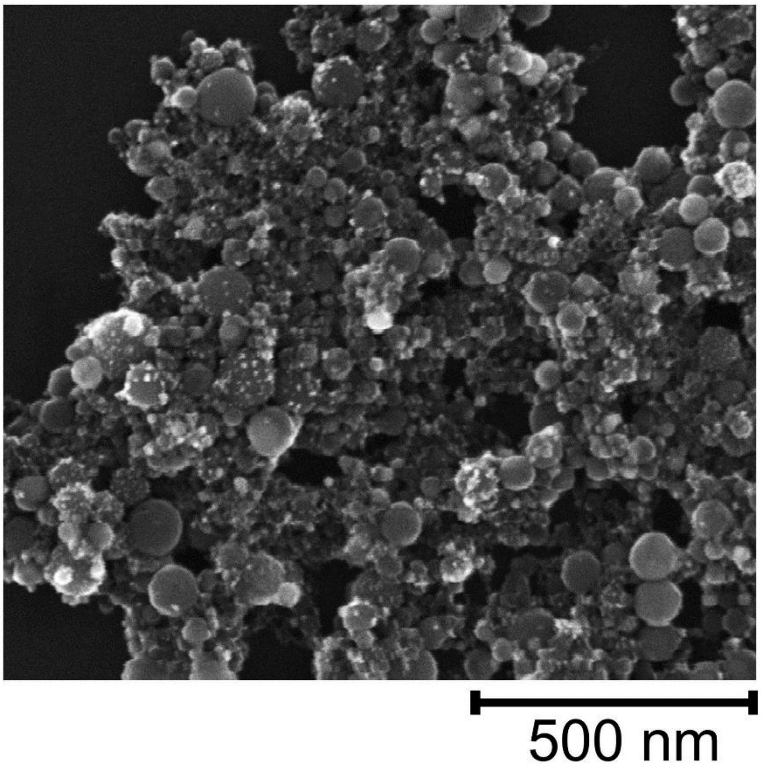

Figure S1. SEM image of the Fe-Au nanoparticles obtained by the method of pulsed laser ablation in liquids. Scale bar: 500 nm.

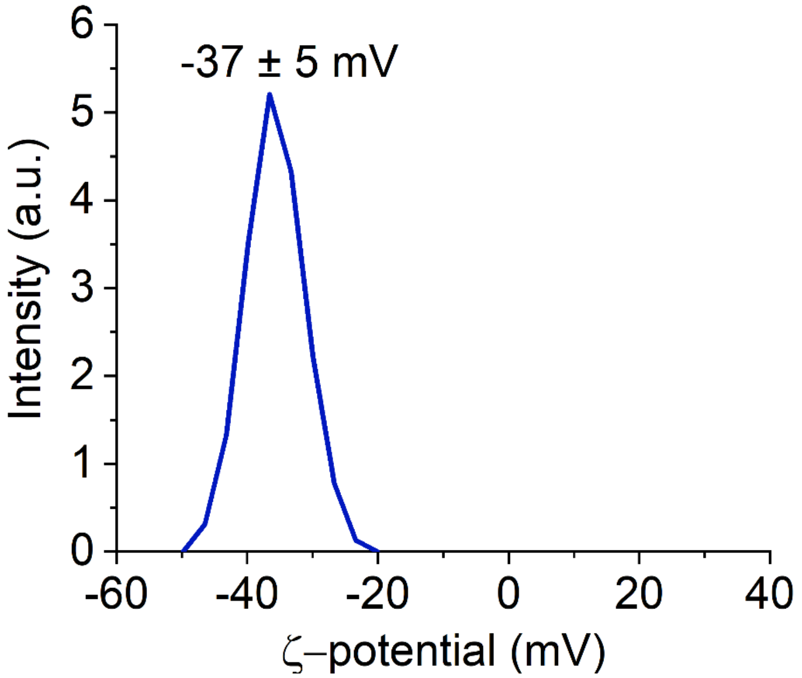

Figure S2. ζ-potential of PAA-coated Fe-Au NPs 25 days after their storage at 4 °C.

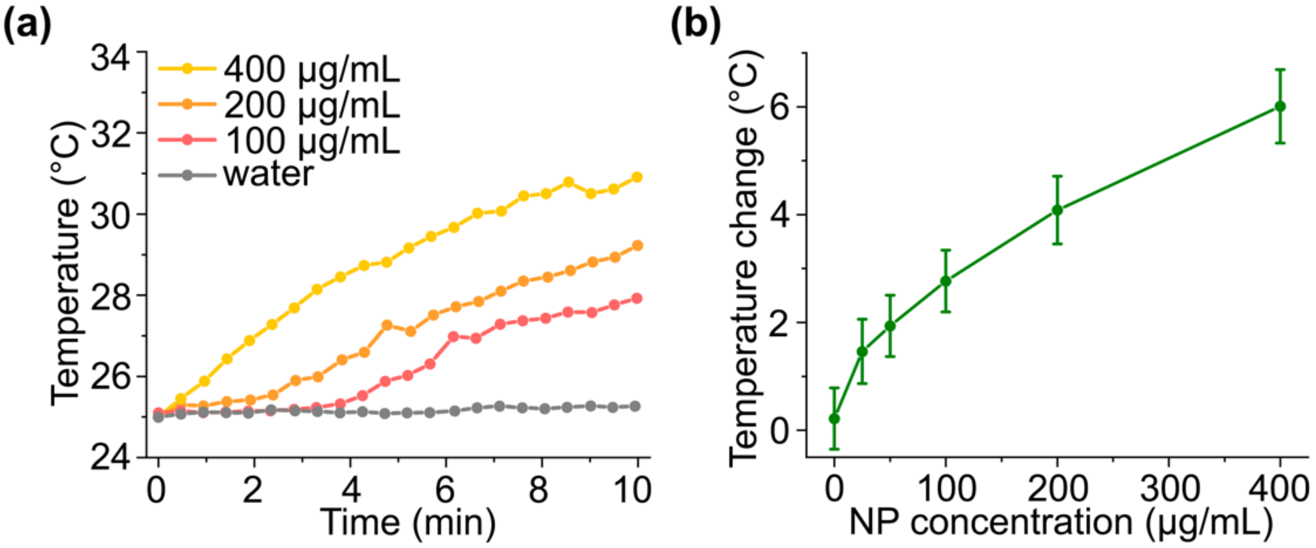

Figure S3. Temperature curves of Fe-Au@PAA NPs (**a**) and dependence of maximal temperature change on the particle concentration (**b**). NPs were irradiated with 100 mW 532-nm laser source for 10 min.

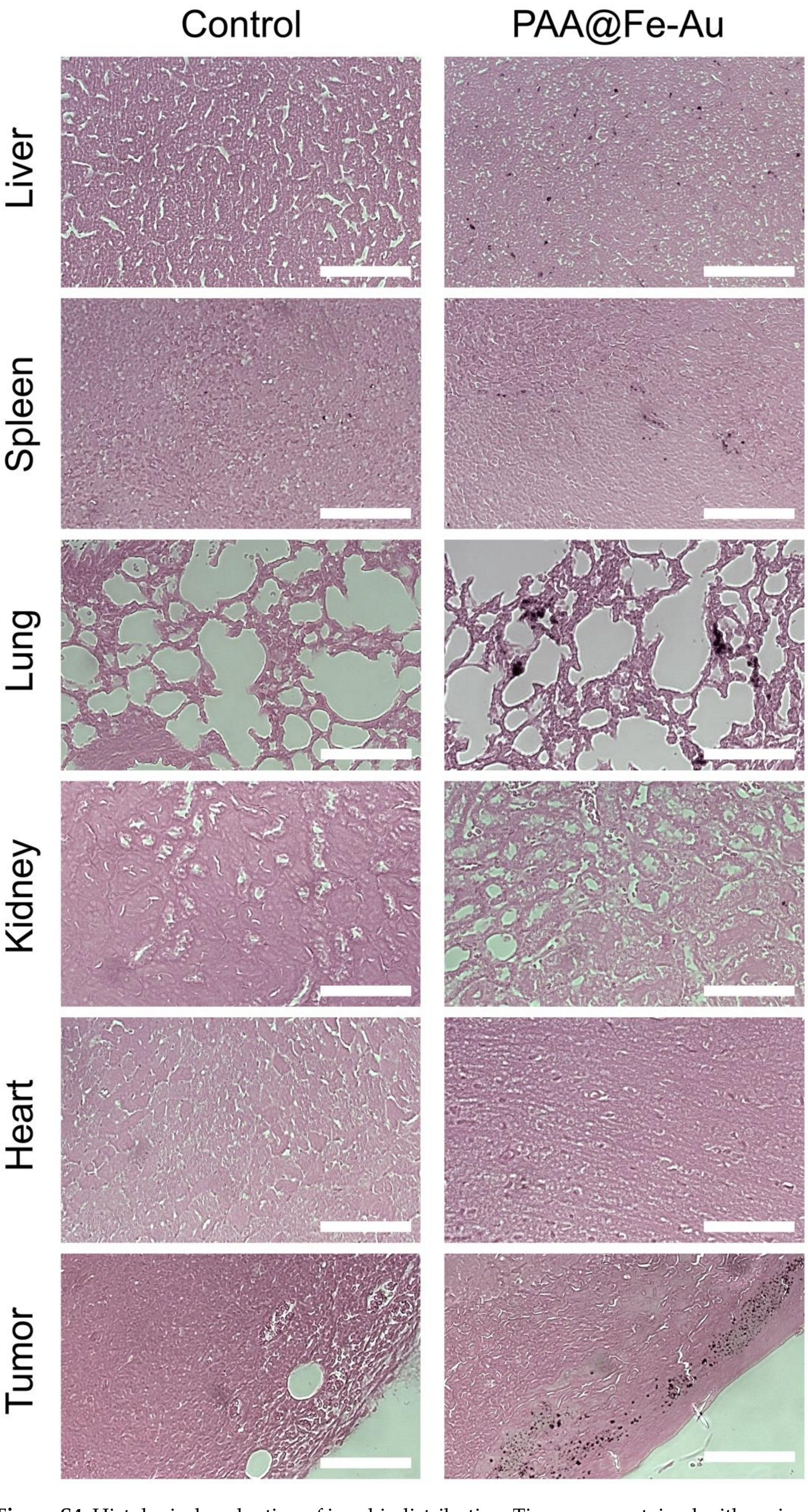

Figure S4. Histological evaluation of iron biodistribution. Tissues were stained with eosin and Perls Prussian blue. Trivalent iron in liver, spleen, lungs and tumor is colored black. Scale bar: 100 μm.
